# Emerging role of rare earth elements in biomolecular functions

**DOI:** 10.1093/ismejo/wrae241

**Published:** 2024-12-08

**Authors:** Wenyu Yang, Kaijuan Wu, Hao Chen, Jing Huang, Zheng Yu

**Affiliations:** Human Microbiome and Health Group, Department of Parasitology, School of Basic Medical Science, Central South University, Changsha, Hunan 410013, China; Human Microbiome and Health Group, Department of Parasitology, School of Basic Medical Science, Central South University, Changsha, Hunan 410013, China; Human Microbiome and Health Group, Department of Microbiology, School of Basic Medical Science, Central South University, Changsha, Hunan 410013, China; Human Microbiome and Health Group, Department of Parasitology, School of Basic Medical Science, Central South University, Changsha, Hunan 410013, China; Human Microbiome and Health Group, Department of Microbiology, School of Basic Medical Science, Central South University, Changsha, Hunan 410013, China

**Keywords:** Rare earth elements (REEs), methanol dehydrogenase XoxF, ethanol dehydrogenase ExaF/PedH, methylotrophs and methanotrophs, lanmodulin (LanM)

## Abstract

The importance of rare earth elements is increasingly recognized due to the increased demand for their mining and separation. This demand is driving research on the biology of rare earth elements. Biomolecules associated with rare earth elements include rare earth element-dependent enzymes (methanol dehydrogenase XoxF, ethanol dehydrogenase ExaF/PedH), rare earth element-binding proteins, and the relevant metallophores. Traditional (chemical) separation methods for rare earth elements harvesting and separation are typically inefficient, while causing environmental problems, whereas bioharvesting, potentially, offers more efficient, more green platforms. Here, we review the current state of research on the biological functions of rare earth element-dependent biomolecules, and the characteristics of the relevant proteins, including the specific amino acids involved in rare earth metal binding. We also provide an outlook at strategies for further understanding of biological processes and the potential applications of rare earth element-dependent enzymes and other biomolecules.

## Graphical Abstract

**Graphical Abstract:**
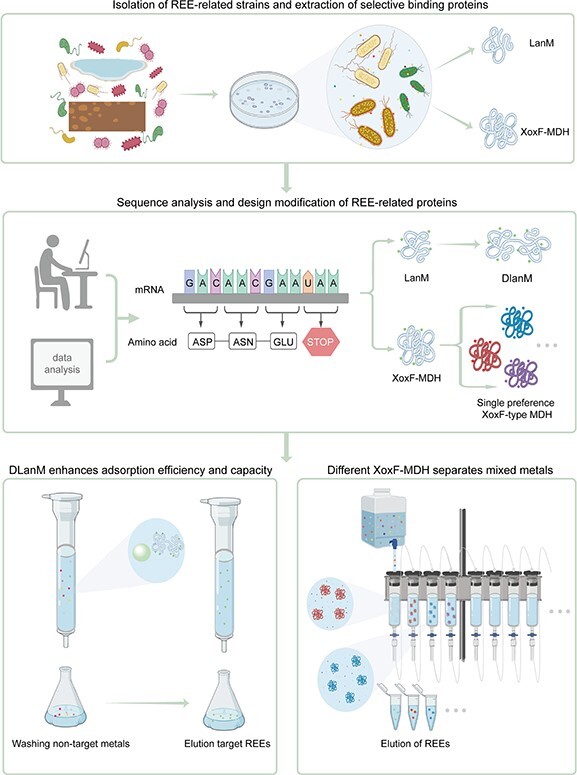

## Introduction

Rare earth elements (REEs) are a group of metallic elements with atomic numbers ranging from 57 to 71. These elements include yttrium (Y^3+^), scandium (Sc^3+^), and 15 lanthanides (Ln^3+^) ranging from lanthanum (La^3+^) to lutetium (Lu^3+^) [1]. REEs are typically categorized into two categories (“light” and “heavy”), although this may be extended to include a third designation (“medium”), based on their relative atomic masses [1, 2].

Despite their misleading name “rare earth,” the average concentrations of REEs in the Earth’s crust are relatively high, reaching up to 0.015% [2, 3], which is comparable to, e.g. concentrations of copper [2, 3]. REEs are highly valued strategic resources due to their widespread use in science and technology, military applications, and other industries. The demand for praseodymium (Pr^3+^), neodymium (Nd^3+^), and dysprosium (Dy^3+^), in particular, is expected to exceed the current supply, based on the existing extraction technologies [4]. At the same time, due to their relatively similar exterior atomic electron configuration and the nucleoid nature of the 4f orbitals, REEs tend to be mixed in the naturally occurring minerals and, thus, it is difficult to separate them from each other [5]. The traditional hydrometallurgical liquid–liquid extraction process for producing purified REEs involves the use of organic solvents, leading to high energy consumption and significant environmental impacts due to the use of harsh chemicals [6, 7]. Biological extraction and separation of target REEs, using microbes or biomolecules, presents an attractive alternative platform [8, 9], which may revolutionize the field [8, 9]. The first evidence on the biological function of REEs was presented in 2011 by demonstrating the activity of a XoxF-type methanol dehydrogenase (MDH) with lanthanides [10, 11]. Subsequently, Ln^3+^-dependence was confirmed for other enzymes such as ethanol dehydrogenases (EDH) [12, 13]. Furthermore, REE-binding capacity was demonstrated for other proteins, namely LanM [14, 15], lanpepsy (LanP) [16], a metallophore methylolanthanin (MLL) [17], and small molecule chelating agents [18, 19]. These discoveries established REEs as biologically active metals, initiating a new and now rapidly expanding field of research.

Using REE-binding proteins for the biological extraction and separation of target REEs provides a practical and viable approach [14]. The distinct crystal structures of these proteins would be expected to provide valuable insights into REE separation, mechanisms of action, and bionic design tactics. Currently, the knowledge on the structure of REE-binding proteins is very limited. In this review, we first summarize recent findings on REE-binding proteins, then we focus on the unique amino acid composition and the 3D structures of several well-characterized proteins. Finally, we highlight emerging technologies in the REE-based biology. This review underscores some of the major directions in the REE research field, to contribute to the existing body of literature.

## Research progress in REE-dependent alcohol dehydrogenases

### Discovery and characterization of REE-dependent alcohol dehydrogenases

Methane and methanol are some of the most abundant organic compounds in the Earth’s atmosphere, and they serve as essential sources of carbon and energy for certain microorganisms, named methylotrophs [20]. One key enzyme that these bacteria use is MDH, catalyzing the conversion of methanol to formaldehyde [21]. The most well-studied enzyme for this conversion, over decades, has been the MxaFI type that uses pyrroloquinolone quinone (PQQ) and calcium (Ca^2+^) as cofactors. However, the genomes of most methylotrophic bacteria were noted to contain a gene encoding a protein similar to MxaF, named XoxF. This gene has been identified as one of the most persistent signatures of methylotrophy [22–28]. While remaining elusive for some time, the role of this protein was revealed circa 2011, by demonstrating its activity with cerium (Ce^3+^) as a cofactor, in *Bradyrhizobium* sp. MAFF211645 [29]. This discovery, followed by subsequent discoveries including other microbes, have established XoxF as Ln^3+^-dependent MDH, and, importantly, revealed the previously unknown biological function of Ln^3+^ [11, 30].

**Figure 1:**
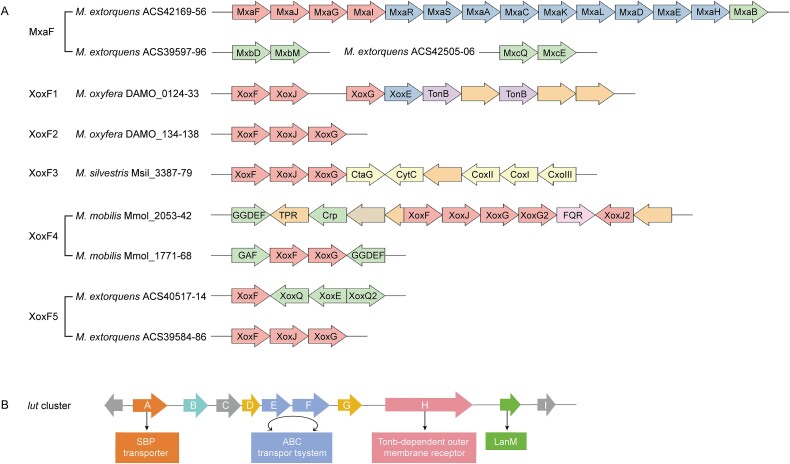
Composition map and functional annotation of *xox* and *lanM* clusters. (**A**) the organization of the MxaFI and XoxF systems of different classes. Genes encoding structural proteins are represented by pink arrows, while auxiliary genes involved in MDH assembly are shown as blue arrows, and regulatory genes are indicated by green arrows. Genes related to methanol metabolism are depicted in various colors. Genes encoding proteins with known or predicted functions unrelated to methanol metabolism are shown in light brown, and the unknown gene is represented in orange. Gene sizes and intergenic regions are not to scale. TonB, tonb-like uptake system; CytC, cytochrome c; cox, cytochrome c oxidase; CtaG, cytochrome c oxidase assembly factor; FQR, putative formaldehyde: Quinone reductase; GAF, GAF-modulated sigma 54-specific transcriptional regulator; GGDEF, diguanylate cyclase; TPR, tetratricopeptide repeat protein; Crp, cyclic AMP-receiving transcriptional regulator; PhoH, PhoH family protein. Species: *M. Extorquens*, *Methylobacterium extorquens* AM1; *M. Oxyfera*, *Candidatus* “Methylomirabilis oxyfera”; *M. Silvestris*, *Methylocella silvestris* BL2; *M. Mobilis*, *Methylotenera mobilis* JLW8. More MDH genome information has been published previously [21]. The gene clusters were mapped using ChiPlot (https://www.chiplot.online/gene_cluster.html). (**B**) Characterization of the lut/lan cluster (MexAM1_META1p1778 to MexAM1_META1p1787). Gene a encodes an SBP transporter, while gene B may encode a group of enzymes that contain carboxylic acid residues adjacent to tryptophan residues in their homologous models. Genes D and G are potentially linked to system function; genes E and F encode components of the ABC transport system; gene H encodes a TonB-dependent receptor protein, and the gray arrows represent unidentified genes. Adapted from [18, 33–35].

Whereas the MxaFI-MDH is a heterotetramer composed of two large subunits (MxaF) and two small subunits (MxaI) [31], XoxF-MDH is a dimer consisting of two XoxF subunits [32]. The MxaF proteins form a tight cluster on the evolutionary tree, whereas XoxF enzymes diverge into at least five major evolutionary branches (Fig. 1A) [21, 36, 37], some of which form sub-branches [21, 38]. For its enzymatic activity in vitro, the MxaFI enzyme requires high pH (pH 9–10) and the presence of ammonia, for reasons that are not fully understood. However, different XoxF enzymes exhibit varying behaviors, where some require both high pH and ammonia and others act at neutral pH without ammonia activation [11, 21, 39, 40].

The mechanisms of regulation of expression of the MxaFI-type MDH have been extensively studied, especially in the model methylotroph *Methylobacterium extorquens* AM1 [41–44]. Genes necessary for the MxaFI function are located in five gene clusters: *mxa, mxb, mxc*, and two *pqq* clusters [45–47]. In contrast, the XoxF-type MDH is encoded by the conserved cluster *xoxFGJ,* with additional copies of *xoxF* present as single genes in the genomes, sometimes in multiple copies and sometimes as parts of different gene clusters [11, 29, 48, 49]. While the XoxF system also requires PQQ as a cofactor, the *mxaACKLD* genes encoding Ca^2+^ insertion and the *mxaRSEH* genes controlling enzyme maturation are not required [21]. The regulation of the XoxF-type MDH expression still requires further studies, and it is expected to be species-specific [11, 21, 50–52]. In many methylotrophs, both MxaFI-type and XoxF-type MDH enzymes are encoded [36, 53]. The meaning and the causes of this redundancy remain poorly understood. Meantime, the field of study of the REE-dependent molecular functions is rapidly developing. In addition to the XoxF enzymes that tend to favor methanol as a substrate, REE-dependent EDH have been characterized in both methylotrophs (*M. extorquens* AM1, named ExaF) and non-methylotrophs (*Pseudomonas putida* KT2440, named PedH), expanding the significance of REEs beyond methylotrophy [12, 54]. The broad distribution of the genes for both ExaF/PedH and XoxF among bacterial taxa has been reported, further highlighting the significance of REE-dependent metabolisms [55]. Furthermore, recently, genes similar to XoxF/PedH were identified in a specific class of marine archaea (HMT), with yet unknown metabolic functions [56].

In conclusion, the growing databases of the REE-dependent alcohol dehydrogenases unequivocally place REEs with other live metals, with important biomolecular functions. As the field of study of the REE-dependent biology progresses, many discoveries are expected in both fundamental and applied areas of REE biological function.

### Properties of REE-dependent alcohol dehydrogenases

The types of metals present in experimental setups affect bacterial growth rate and gene expression profiles [29, 57–59]. Early results on the XoxF-MDH enzyme kinetics (Table 1) indicate that these enzymes not only show a greater methanol oxidation rate but also have a higher affinity for methanol than the MxaFI-MDH enzymes. They also sow different preferences for REEs. Purified XoxF-MDH demonstrated varied activity when model bacteria are cultured with LREEs or HREEs [12, 29, 63, 70, 71]. For example, the growth of *Methlacidiphilum fumariolicum* SoIV was similar with several different LREEs, whereas using HREEs resulted in unstable behavior, reduced growth, and changes in MDH activity/biochemical characteristics [11]. XoxF-MDH from *M. extorquens* AM1 showed higher activity with Nd^3+^ compared to La^3+^ [72]. REE-dependent EDHs also showed a preference for LRREs. When grown in media containing REEs with atomic weights greater than Nd^3+^, the specific EDH activity steadily diminished [12]. LREE-dependent EDHs (ExaF/PedH) demonstrated better catalytic efficiencies than the Ca^2+^-dependent EDHs (ExaA/PedE). This may be due to the more optimal metal coordination in the active site of the enzyme [11, 12, 21, 54, 55]. Furthermore, in the absence of MxaFI or XoxF, ExaF can support growth on methanol, in the presence of LREEs [54, 59]. Kinetic studies indicated that ExaF oxidizes both ethanol and methanol at similar rates (*V*_max_) while the *K*_m_ is much for ethanol [54]. It has been suggested that the metal cofactors in PedH undergo reversible coordination modifications. PedH also appears to be able to coordinate Ca^2+^ when grown in the absence of REEs [11, 12, 21, 54, 55]. In contrast, in XoxF, the cofactor cannot be removed reversibly highlighting the intricacy of the cofactor-binding specificities among different REE-dependent enzymes [18, 69, 73]. Many parameters, including metal ion radius, ligand preference, ligand exchange rate, substrate orientation, and purification efficiency are thought to influence the activity of REE-dependent enzymes. To better understand the activity and function of these enzymes, it would be important to integrate kinetic data, crystallography, and differential electrochemical mass spectrometry (DET) studies.

**Table 1:** Summary of Kinetic parameters and metal-binding sites for REE-dependent alcohol dehydrogenases obtained from previously published data, including comparative data for MxaF-MDH.

Organism	Enzyme	*K*_m_ (μM)	Metal-dependent	PDB ID	Metal binding sites	Reference
*Methylorubrum extorquens*	MxaF	–	Ca^2+^	1H4I	Glu177, Asn261	[60]
*Methylorubrum extorquens*	MxaF	3	Ca^2+^	1W6S	Glu177, Asn261	[61, 62]
*Methylacidiphilum fumariolicum* SoIV	XoxF	3.6 ± 0.4	Eu^3+^	6FKW	Glu172, Asn256, Asp299, Asp301	[63]
*Methylorubrum extorquens* AM1	XoxF1	–	La^3+^	6OC6	Glu192, Asn276, Asp318, Asp320	[13]
*Methylacidimicrobium thermophilum* AP8	XoxF1	1.4 ± 0.6	Nd^3+^	7O6Z	Glu204, Asn290, Asp345, Asp347	[64]
*Methylobacterium aquaticum* Strain 22A	XoxF1	3	La^3+^	–	–	[65]
*Thermophilic Methylothermalis* aethiopiae(LS7-MT)	XoxF1	–	–	–	–	[66]
*Methlacidiphilum fumariolicum* SoIV	XoxF2	0.8 ± 0.3	La^3+^-Nd^3+^	4MAE(Ce^3+^)	Glu172, Asn256, Asp299, Asp301	[11]
*Methylotenera mobilis* JLW8	XoxF4–1	55 ± 32	La^3+^-Dy^3+^	–	–	[38]
*M. mobilis* JLW8	XoxF4–2	42 ± 18	La^3+^-Sm^3+^	–	–	[38]
*Methylomonas* sp. LW13	XoxF5	39 ± 11	La^3+^-Nd^3+^	–	–	[38]
*Bradyrhizobium* sp. MAFF 211645	XoxF5	29	Ce^3+^	–	–	[29]
*Methylomicrobium buryatense* 5GB1	XoxF5	–	La^3+^	6DAM	Glu197, Asn285, Asp327, Asp329	[67]
*Bradyrhizobium diazoefficiens* USDA 110	XoxF5_B.d.	15 ± 2	La^3+^-Nd^3+^	–	–	[55, 68]
*Grimontia marina* CECT 8713	XoxF5_G.m.	24 ± 2	La^3+^-Nd^3+^	–	–	[55]
*Methyloversatilis* sp. FAM1	XoxF5_M.d.	15 ± 2	–	–	–	[55]
*Methylorubrum extorquens* AM1	XoxF5_M.e.1	21 ± 1	La^3+^-Nd^3+^	–	–	[55]
*Methylorubrum extorquens* AM1	XoxF5_M.e.1	44 ± 5	La^3+^-Nd^3+^	–	–	[69]
*Methylorubrum extorquens* AM1	XoxF5_M.e.2	12 ± 2	La^3+^-Nd^3+^	–	–	[55]
*Rhodovulum kholense* DSM 19783	XoxF5_R.k.	17 ± 1	La^3+^-Nd^3+^	–	–	[55]
*Sinorhizobium meliloti* 5A14	XoxF5_S.m.	23 ± 2	La^3+^-Nd^3+^	–	–	[55]
*Tistlia consotensis* DSM 21585	XoxF5_T.c.	30 ± 6	La^3+^-Nd^3+^	–	–	[55]
*Methylopila* sp. M107	ExaF/PedH_M.s.	16 ± 3	Ln^3+^-Yb^3+^	–	–	[55]
*Methylorubrum extorquens* AM1	ExaF	0.9	La^3+^	–	–	[54]
*M. aquaticum* Strain 22A	ExaF	–	La^3+^	–	–	[65]
*Pseudomonas putida* KT2440	PedH	177	La^3+^-Tb^3+^	6ZCW(Pr^3+^)	Glu199, Asn281, Asp323, Asp325	[12]

### Structural characteristics of REE-dependent alcohol dehydrogenases

**Figure 2:**
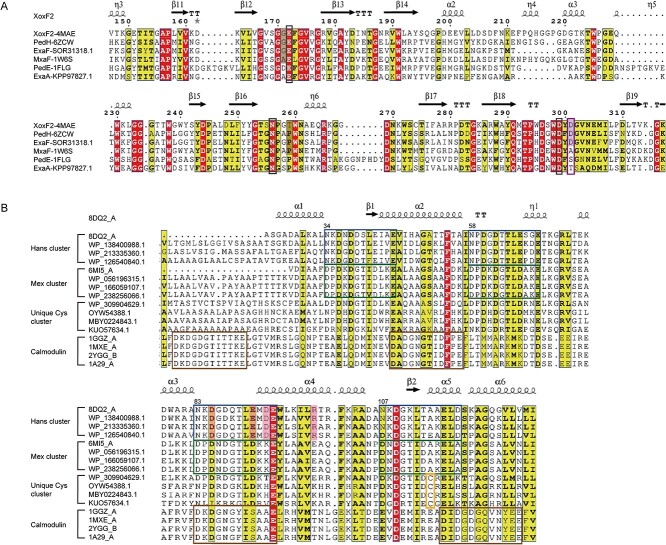
Multiple sequence alignment analysis of alcohol dehydrogenases and LanMs. (**A**) Amino acid sequence comparison results of alcohol dehydrogenase. Black boxes indicate conserved metal catalytic sites and purple boxes indicate special metal binding sites. The special amino acid substitution of XoxF2 and MxaFI is shown in the black shade. Protein sequence information can be accessed using the NCBI reference sequence and PDB ID provided in the fig. (**B**) Amino acid sequence comparison results of LanM. Four categories of LanM are shown, listed from top to bottom: Hans, Mex, unique Cys, and Calmodulin cluster sequences. The four EF motifs involved in metal binding are highlighted with differently colored boxes. Yellow boxes indicate specific and conserved Cys residues. The pink vertical bars in Hans-LanM represent amino acid residues involved in a specific hydrogen bond network (for more information see Fig. 5B). Mex-lanM PDB ID: 6MI5_A; Hans-lanM PDB ID: 8DQ2_A. Other protein sequence information can be accessed using the NCBI reference sequence provided in the figure. Multiple sequence alignment analysis was performed by using Clustal omega (http://www.ebi.ac.uk/Tools/msa/clustalo/) and ESpript v.3.0 server [75].

**Figure 3:**
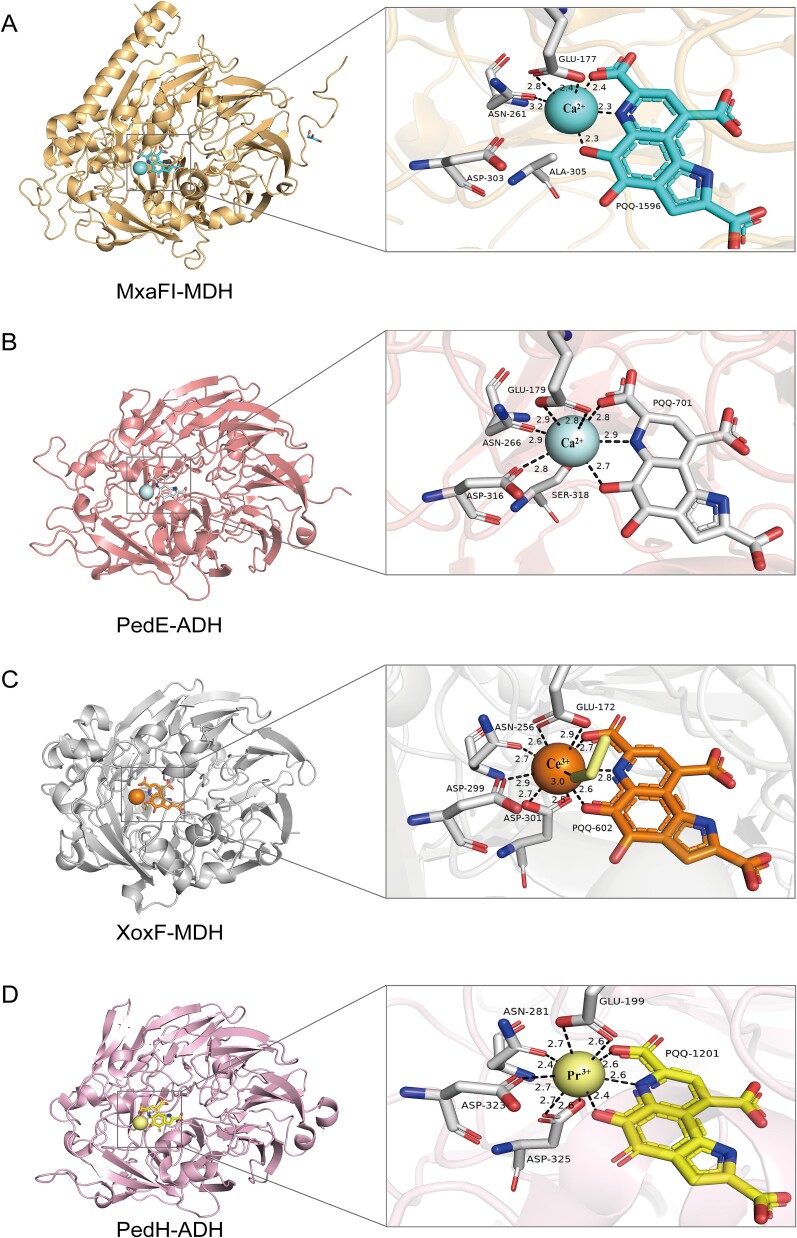
A comparative depiction of the 3D structure of alcohol dehydrogenases. (**A**) Three-dimensional structure of MxaFI-MDH, PDB ID: 1W6S. Black bonds indicate ligand bonds, distances in Å, where the Asn261 ligand bond exceeds three Å and is not shown; (**B**) 3D structure of PedE-EDH, PDB ID: 1FLG. Black bonds indicate ligand bonds and distances are in Å. To highlight the specificity of asp, Asp316 ligand bonds are shown even though they are longer than three Å; (**C**) three-dimensional structure of XoxF2-MDH, PDB ID: 4MAE. Black bonds indicate coordination bonds and distances are in Å. The involvement of Asp301 in metal coordination can be notably found, resulting in an increased number of coordinations and, unlike Asp299, bidentate coordination; (**D**) 3D structure of PedH-EDH, PDB ID: 6ZCW black bonds indicate coordination bonds, distance unit is Å. It can be significantly found that Asp325 is involved in metal coordination, leading to an increased number of coordination, and is different from Asp323, and is bidentate. Combined with the difference in Fig. 3—Asp301 sequences, it can be seen that REEs-MDH/EDH have specific asp coordination bonds, forming a more robust binding pocket. The protein structure and active site were displayed using Pymol-software [74].

While significant progress has been achieved in studying REE-dependent alcohol dehydrogenases, a further level of knowledge is required to better understand the biological function and the underlying mechanisms of REE involvement. Therefore, elucidating the unique differences in structure among Ca^2+^ and REE-dependent proteins is imperative. The first breakthrough came with the resolution of the XoxF-MDH crystal structure in *M. fumariolicum* SolV, revealing the presence of the REE ions within the catalytic site, one of the coordinating amino acids being aspartate (Asp301) that is not present in the MxaF proteins [11]. Asp301 is conserved in the sequences of all XoxF proteins (Fig. 2A). Other amino acid residues proposed to be involved in RRE coordination are Gly171 that replaces Ala171 in MxaF enzymes, and Thr259 that replaces Pro259 (Fig. 2A**,**  Fig. 3A, C). The former is proposed to assist in coordination of the larger REEs, and the latter is proposed to assist in coordination of the smaller REEs. In the MxaF, Pro259 is proposed to affect how the protein skeleton is positioned spatially and to help Asn256 in coordination of Ca^2+^ (Fig. 2A**,**  Fig. 3A, C) [11].

Similarly to XoxF-MDH, the known ExaF/PedH EDH enzymes have an additional Asp residue in place that is usually occupied by Ser or Thr in ExaA/PedE-type EDHs. The crystal structure of PedH from *P. putida* KT2440 has been determined with Pr^3+^, demonstrating that this additional Asp residue is involved in coordinating the metal, thus suggesting an important role in the conformation and the function of PedH (Fig. 2**,**  Fig. 3B, D) [11, 21]. Thus, the crystal structures of both XoxF and PedH emphasize the importance of additional Asp residues for REE coordination and overall enzyme function [21, 54, 67, 70, 76, 77]. This makes Asp a signature aiding in the identification of REE-dependent proteins (more on this below). Furthermore, distinctive signature residues exist among different XoxF-MDH types. For instance, an additional aspartic acid residue Asp172 appears to be characteristic of the XoxF1-type MDH while the corresponding amino acid in other XoxF enzymes (XoxF2-XoxF5) is Gly [64]. While all the quinoproteins encoded by the HMT genomes lacked the conserved disulfide cysteine-cysteine motif typical of all true MDHs [78, 79], four variants contained the Asp-Tyr-Asp (DYD) motif common to the REE-dependent alcohol dehydrogenases [21, 56]. Of course, the function of these hypothetical quinoproteins remains completely unknown.

It is important to take into account the fact that distinct bacterial strains expressing XoxF and ExaF/PedE proteins exhibit varying preferences for distinct REEs, indicating particular affinities for particular REEs. However, the metal binding sites of these alcohol dehydrogenases are conserved in terms of the active amino acids, with DYD being the signature. It is hypothesized that the various sizes of the metal active site pocket, the diverse hydrogen bonding forces between the metal and the specific amino acid residues, and even the distinct helix stems created by the multiple amino acids surrounding the metal binding sites are responsible for metal preferences and activity. The more careful determination of these distinctions is one direction for the fast-developing field of REE biology.

## Advances in the identification and characterization of REE-binding proteins

### Discovery and characterization of LanM

To ensure the correct and efficient metallization of enzymes within the cell, many precedents for metalloenzyme systems have been proposed [80]. A protein of ~12 kDa, named LanM (Mex-LanM), was copurified with XoxF protein from *M. extorquens* [15], suggesting that it might be functionally connected to XoxF-MDH. LanM contains four carboxylate-rich metal–ligand motifs known as the EF-hands. This strongly suggested that REE ions must be selectively recognized and transported into cells [14, 15]. LanM possesses several key features. First, each EF-hand fragment contains 12 residues of metal-binding rings rich in carboxylic acids, which are flanked by alpha-helixes. Second, the spacing between the adjacent EF-hands in LanM is 12 to 13 residues, in contrast to the 25 residues typically found in the Ca^2+^-responsive EF-hand proteins (Fig. 2B). This arrangement results in an atypical triple helix bundle structure with metal binding sites located on the periphery. Third, the presence of Pro residues in the second position of at least one EF-hand in LanM is a distinct feature that may potentially impede responses to Ca^2+^ and other metal ion interactions, thus ensuring metal selectivity [15]. LanM exists as an inherently disordered protein in the apolipoprotein state. Although the secondary structure of LanM is less pronounced than that of other EF-hand-containing proteins, in the presence of REE ions, LanM can undergo a considerable, coordinated conformational transition to a compact conformation with ~50% helical structure [14, 15]. At the same time, LanM exhibits weak responsiveness to metal ions other than REEs, requiring concentrations of ions such as Ca^2+^ at the millimolar level [14, 33]. A special feature of LanM is different affinity for LREEs and HREEs [18], with the conformational reaction of LanM favoring the LREEs over the more acidic HREEs [6]. A second LanM protein was identified in *Hansschlegelia quercus* and named Hans-LanM, and it also showed preference for LREEs [14]. Hans-LanM exhibits significant sequence divergence with the Mex-LanM (33% sequence identity), including differences in metal binding sites. In laboratory experiments, Hans-LanM could efficiently accomplish single-phase baseline separation of Dy^3+^ from Nd^3+^, with a purity level of more than 98% and a yield of more than 99%, thus being more selective than Mex-LanM (Table 2). In addition, Hans-LanM exhibits high structural stability even at high temperatures, up to 95°C, and maintains its REEs-binding capacity under acidic conditions (pH 2.5) [81]. This remarkable stability highlights the potential for LanM proteins in enriching REEs for industrial purposes, especially those proteins that may be originating from acidic environments such as mine drainage sites. Because of the aforementioned superior qualities, Hans-LanM has become an early model for biological extraction and separation of REEs [18, 81, 84].

**Table 2:** Apparent dissociation parameters and metal sites of lanmodulin (LanM) derived from previously published data (Recorded only with relatively complete parameters).

Organism	Protein	*K*_d,app_ (pM)	Metal-dependent	PDB ID	Reference
*Hansschlegelia quercus*	Hans-LanM	68	La^3+^	8DQ2	[14]
*Hansschlegelia quercus*	Hans-LanM	91	Nd^3+^	–	[14]
*Hansschlegelia quercus*	Hans-LanM	2600	Dy^3+^	8FNR	[14]
*Methylorubrum extorquens* AM1	Mex-LanM	–	La^3+^	8FNS	[14]
*Methylorubrum extorquens* AM1	Mex-LanM	70 ± 10	Pr^3+^	–	[81]
*Methylorubrum extorquens* AM1	Mex-LanM	100 ± 10	Gd^3+^	–	[81]
*Methylorubrum extorquens* AM1	Mex-LanM	200 ± 50	Dy^3+^	–	[81]
*Methylorubrum extorquens* AM1	Mex-LanM	260 ± 60	Ho^3+^	–	[81]
*Methylorubrum extorquens* AM1	Mex-LanM	177	Y^3+^	6MI5	[82]
*Methylobacterium aquaticum* Strain 22A	His-LanM	–	La^3+^	–	[83]

The *lanM* gene in *M. extorquens* AM1 is part of a gene cluster suggesting that the neighboring genes may be responsible for the uptake and transport of REEs, and this hypothesis was tested via a transposon mutagenesis screen [34]. The group of genes identified, named as “*lut*” or “*lan*” [33, 35], may be critical to our understanding of the uptake and transport processes critical to REE-based catalysis (Fig. 1B) [18]. Knockdown tests have verified some of these key genes, including the ones for a TonB-dependent receptor protein (LutH), the ATP-binding box (ABC) transporter (LutEF), and the substrate binding protein (SBP) of the transporter (LutA), which were responsible for intracellular transport of REEs [34, 35]. La^3+^-Nd^3+^ were shown to be selectively transported into the cytoplasm of *M. extorquens* AM1 with the help of LanM [81]. However, Fujitani et al. discovered that, while *lanM* is linked with additional specific *lut* genes in the genomes of *M. extorquens* AM1 and *Methylobacterium extorquens* PA1, it is linked with different genes in the genome of *Methylobacterium aquaticum* Strain 22A [83]. Phenotypes were not identified in *lanM* mutants of *M. extorquens* PA1 or *M. aquaticum* Strain 22A suggesting that alternative REE sensors may exist [34, 35, 83].

**Figure 4:**
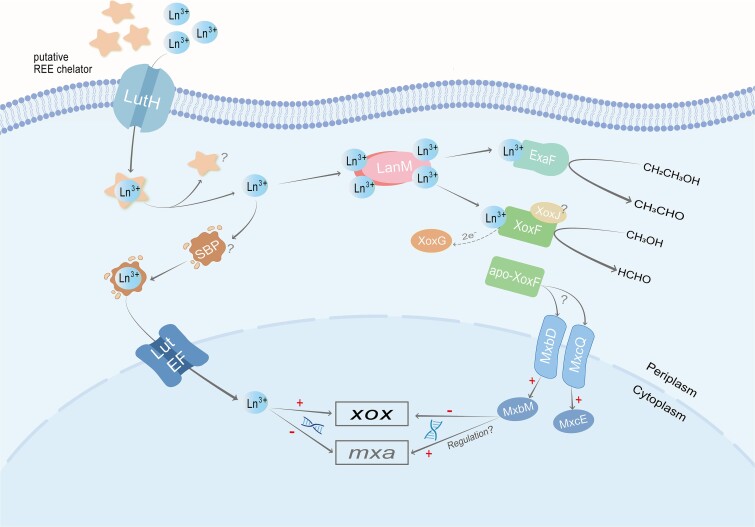
Schematic diagram of the transport model for REEs uptake in *M. Extorquens***. I**n the case of *M. Extorquens*, alcohols in the periplasm can be oxidized to aldehydes by REEs-dependent XoxF-MDH/ExaF-EDH. The electrons produced by these reactions are transferred from these enzymes to their dedicated cytochrome XoxG. For further metabolism, the aldehydes produced should be transferred to the cytoplasm. The functions of XoxF proteins include enzymatic oxidation of methanol in the periplasm and involvement in the regulation of other genes, possibly through the sensing or transduction of extracellular signals (REEs and the presence of stress factors). The question mark “?” indicates that there is no definite conclusion or that the intracellular pathway is unknown. The plus sign “+” represents positive regulation, and the minus sign “-” represents negative regulation. Lut corresponds to the gene shown in Fig. 1A, based on the text description. Adapted and updated from [18, 35, 65, 85, 86].

The discovery and characterization of the first LanM protein, importantly, expanded the range of known REE-binding proteins beyond REE-dependent alcohol dehydrogenases. LanM protein exhibited high selectivity and binding efficiency for REEs, making it an excellent candidate for extracting pure REEs. To address the needs of society and the industry for the separation and enrichment of REEs, it would be of great interest to fully utilize this protein’s unique biological function. It is unclear if LanM is involved in REE transport, but it may do so to some extent and under certain circumstances (Fig. 4**).** However, some important questions remain unanswered, such as whether the assumed chelating agent that allows REEs to penetrate the cell membrane exists, and what would be the fate of these hypothetical proteins after their function is completed. How does the SBP work with the ABC transport system to help REEs enter the cytoplasm? While the REE ions appear to enter the periplasm via the TonB-dependent receptor protein LutH, there is no experimental evidence that free REEs existed in the periplasm. The need for REEs to enter the cytoplasm is unknown. If REEs play a regulatory role in the cytoplasm and other, unknown critical proteins are present in the cytoplasm for such regulation, the requirements for cytoplasmic input and output may be different. There is still a lot to discover about the role and the mechanism of action of the LanM protein.

#### Structural characteristics of LanM

**Figure 5:**
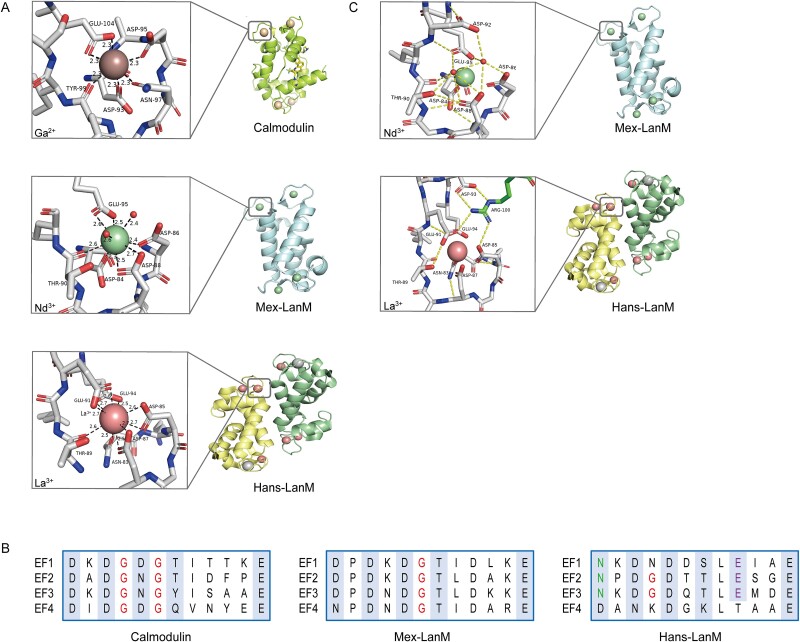
Comparison of metal-binding sites, unique hydrogen bond networks, and EF motifs in Mex-LanM and Hans-LanM**. (A).** 3D structure of Calmodulin, Mex-LanM, and Hans-LanM showing coordination bonds (all EF3 interfaces), with PDB IDs: 1GGZ, 8FNS, and 8DQ2, respectively. The black bonds denote coordination bonds, with distances in Å. The common change when comparing the three is unique in the coordination of asp. (**B**). A visual comparison of the four EF motifs Calmodulin, Mex-LanM, and Hans-LanM. The Calmodulin EF-hand motif features highly conserved Gly residues at positions 4 and 6. In contrast, the EF-hands of Hans-LanM and Mex-LanM contain Gly only at positions 4 and 6, respectively. Additionally, Hans-LanM has an Asn(N1) residue at the first position of the EF hand. This amino acid substitution of asp(D1) in Mex-LanM and Calmodulin results in a distinct peptide backbone structure. Moreover, the presence of Glu(E9) in EF1–3 of Hans-LanM is not observed in the other proteins (**C**). Comparison of the hydrogen bonding networks of Mex-LanM, Hans-LanM. The extensive second-sphere interactions at the lanthanide binding sites in Hans-LanM are most prominent in EF3. In Hans-LanM, the dimer interface forms a hydrogen bonding network, where the two carboxylic acid ligands, Asp85 and Glu91, from each monomer in EF3 interact with the other monomer, contributing to the dimer interface. Arg100 plays a key role in reinforcing the bidentate binding mode and is further stabilized by Asp93. These interactions represent the only polar contacts at the dimer interface, providing a mechanism to regulate the size of the lanthanide binding site on EF3. The protein structure and active site were displayed using Pymol-software [74].

The discovery of LanM greatly expanded our understanding of the unique sequence and active site structure of REE-dependent proteins. Notably, distinctions exist not only between Calmodulin (CaM, a Ca^2+^-binding protein) and the two forms of LanM, but also between the two LanM variants. LanMs preserve the important Asp, Asn, and Glu residues within the traditional Ca^2+^-binding EF-hand motif, where specialized Asp residues aid in REE binding (Fig. 5A, B). In Mex-LanM, the coordination number increase is attributed to the bidentate coordination of D (Asp) 5 and an additional solvent ligand [15], whereas in Hans-LanM, the coordination number increase is initially marked by the absence of the solvent ligand and the bidentate coordination of D3 alongside an additional E (Glu) nine residue [14]. Furthermore, while CaM has highly conserved Gly residues at the 4th and 6th positions of each EF-hand, Hans-LanM, and Mex-LanM EF-hands have Gly at only one of these positions (Fig. 5B). Moreover, in Hans-LanM, N (Asn) 1 occupies the first place in the EF-hand, but in Mex-LanM and CaM, it is D1. Differences in amino acid composition can cause changes in peptide backbone structures, affecting helical conformations and interacting amino acids, similarly to the multistage effects seen in metal binding sites and their surroundings. While the particular process is unknown, it is possible that the presence of extra aspartic acid coordination accounts for LanM’s 10^8^-fold improvement in REE selectivity over Ca^2+^ [15].

Given the reported differences in L/HREE *K*_d_ values between Hans-LanM and Mex-LanM (Table 2), a thorough examination of the structural differences between these LanM variants was carried out in attempt to find causes other than differences in amino acid sequences. Hans-LanM’s EF3 has a separate dimer interface defined by an extensive hydrogen bond network involving numerous ligands, which is not present in Mex-LanM. Notably, specific EF3 residues in each monomer form hydrogen bonds with Arg100 in the opposite monomer (Fig. 5C), which strengthens the dual-toothed binding motifs of Asp85 and Glu91. Hans-LanM’s EF-hand 9th Glu residue and Asp93 (EF3-D11), which provides additional support for Arg100, are both found only in Hans-LanM (Fig. 5B, C). While the metal sites of Mex-LanM exhibit similar hydrogen bonding patterns, solvent molecules H_2_O1(w1) and H_2_O2 (w2) substituted for Glu69, while no analogous amino acid residues were found around the metal binding sites observed in Mex-LanM. This may imply that metal binding may be governed by site occupancy and coordination geometry. However, even after the hydrogen bond network was removed, Hans-LanM did not alter the affinity difference between LREEs and HREEs. Mattock et al. conducted a comparison of the crystal structures of these complexes and found that the discrepancy primarily stemmed from the low affinity and synergistic properties of the HREE complexes, with minimal changes in the primary conformation. While the hydrogen bond length at the metal binding site underwent alteration, a shift in carboxylic acid also occurred within the specialized hydrogen bond network, resulting in a change in the network’s length [14]. Consequently, there was a reduction in affinity, coordination numbers, and metal equivalents across different chains. However, the mechanism behind these structural alterations remains unclear, with only a speculation that they impact metal coordination. Additionally, Mattock et al. mention a group of LanMs containing two Cys residues, one near EF1 and the other near EF4 [14]. As LanM is a periplasmic protein, these residues likely form disulfide bonds, potentially offering additional structural stability to LanM. This hypothesis would require experimental support [14].

According to the current limited crystal structure data, while it is impossible to explain the true causes for metal selectivity, as well as for differences in the affinity for LREEs and HREEs, it is certain that differences in hydrogen bonding may affect the overall stability of the protein folding state in the REE-LanM complexes. Furthermore, the coordination number of LREEs and HREEs, the number of metal accommodations in the protein chain, and the alterations in amino acids near the metal binding sites, as noted above, would affect the backbone structure. These are the likely explanations for the discrepancies observed. Perhaps, the metal selectivity of LanM proteins could be attributed to the unique stacking effect of LanM. In the future, it would be important to collect all aspects of structural data and investigate and verify whether the selective difference between LREEs and HREEs may be achieved by structural variations.

### Discovery and characterization of REE-binding protein Lanpepsy

At present, there are still major gaps in understanding the uptake, utilization, and storage of lanthanides by microorganisms. However, it is already clear that there must be multiple mechanisms for REE utilization by different microbes, and these processes must involve biomolecules with different functions. Hemmann et al. found a 19 kDa periplasmic protein of unknown function in an obligate methylotroph *Methylobacillus flagellatus* by histological methods, and they named it LanP. This protein contains two typical PepSY domains. It was the first member of the PepSY family to bind REEs [16]. Although LanP shows little sequence or structure similarity to LanM, its metal-active site contains negatively charged glutamate and aspartic acid residues, similarly to LanM [[5, 82]. LanP has up to four Ln^3+^ binding sites, with remarkable binding affinity that exceeds even that of the metal-chelating dye Arsenazo III (AZ3) [16]. Isocalorimetric (ITC) measurements confirmed that LanP was able to bind lanthanides (Ce^3+^, La^3+^, Nd^3+^, Y^3+^, Pr^3+^) and also exhibited binding to Ca^2+^. Since *M. flagellatus* does not encode LanM, LanP likely replaces its function. It should be noted that the LanP, under the test conditions, did not appear to be directly connected to the upregulation of XoxF expression or the uptake of Ln^3+^, nor did it directly affect the growth of the bacterium [16]. Although more detailed analysis is needed to fully understand the dynamics of LanP binding to Ln^3 +^, the above evidence suggests that LanP has the potential to be employed in industrial REE separation.

### Discovery and characterization of lanthanide chelator Methylolanthanin

To adapt to environmental conditions, metal chelators are often secreted by bacteria, enabling an increase in metal bioavailability [87]. In a study by Zytnick et al., a low-solubility Ln^3+^ source (Nd_2_O_3_) was utilized to discover and structurally characterize the first known metallophore used for Ln^3+^ acquisition, named MLL [17]. MLL represented a novel metallophore. It has been demonstrated that MLL is capable of binding Ln^3+^ of varying sizes such as La^3+^, Nd^3+^, and Lu^3+^ [17]. Overexpression of MLL biosynthetic genes enhanced growth with REEs and REE bioaccumulation, while deletion of these genes resulted in significant defects in Ln^3+^ bioaccumulation. However, the presence of MLL was not obligatory [17]. Notably, MLL differed significantly from other metallophores by containing a characteristic 4-HB fragment, a feature not reported in previously characterized metallophores. This structural distinction suggested an advantageous evolutionary adaptation to efficiently acquire Ln^3+^ for cellular needs [17]. The existence of alternative systems for REE uptake highlights the importance of Ln^3+^ in microbial metabolism and warrants further research to elucidate the intricate relationships between growth and REE storage.

## Research progress in understanding regulation by REEs

In the absence of Ln^3+^, methylotrophs grow on methanol by utilizing the Mxa system reliant on Ca^2+^ [59, 88, 89]. However, in the presence of REEs, the expression of the Mxa system is repressed and the XoxF system is activated. This phenomenon was termed the REE switch [57, 88, 90]. This regulation happens in a dose-dependent fashion. In cases where both enzymes are present, both can be expressed at the same time [35, 54, 58, 59, 89–91]. Current data suggest that two-component systems comprised of membrane-binding sensor kinases and cytoplasmic response regulators are involved in regulating the expression of MDH enzymes [92]. In *M. extorquens* AM1, two such regulators MxbDM and MxcQE are known (Fig. 4) [93]. When REEs are not present, the response regulator MxbM appears to be required to inhibit the expression of the Xox system [94]. MxbD, a sensor kinase, was also shown to be required for MxaF expression and XoxF1 inhibition in *M. aquaticum* Strain 22A [65]. At the same time, XoxF itself appears to serve as an activation signal detected by MxcQ, hence triggering the expression of the *mxa* gene cluster [59]. However, in *M. flagellats*, the protein levels for two two-component regulation systems show no change after the addition of La^3+^ [16]. XoxF is also thought to contribute to peripheral REE sensing, resulting in diminished *xox* inhibition, and in *mxa* downregulation, via interactions with MxcQ, MxbD, or potentially both [59, 94]. However, due to differences in the proteomic composition of different species and strains, the regulatory systems may vary significantly.

*Methylomicrobium buryatense* 5GB1C lacks homologs of *mxbDM* or *mxcQE* but it encodes a sensor kinase named MxaY, which is predicted to bind small molecules, and this protein was suggested to play a role in detecting “free” REEs [88]. A putative REE-binding protein was implicated in activating the orphan response regulator MxaB in the absence of REEs, which would cause *mxa* expression to be induced and *xox* to be suppressed [88]. In addition, the presence of histidine kinase gene MxaY could regulate the MxaB gene and promote the stable expression of MxaB and MxaF, suggesting that MxaY is involved in controlling the activation and inactivation of the alternative MDH systems [95]. Curiously, in one organism, Ce^3+^ had less impact on MxaFI expression when copper was present, suggesting that Copper (Cu^2+^) may also be involved in the REEs switch [57, 89, 90, 96]. However, such regulatory pattern was not reported for other species [57, 89–91, 96].

PedR2/S2 two-component system was identified and implicated in the regulation of PedH in *P. putida*, the REE-dependent EDH, and PedE, Ca^2+^-dependent-EDH [97]. Moreover, a similar MxaY/MxaB-type system was found to regulate the switch between PedH/PedE enzymes [12, 97, 98]. However, the exact mechanism by which the switch is governed remains unknown and requires further research. It should also be noted that a complex interplay may exist between the different regulatory factors, and this network is yet to be fully understood [99, 100].

In conclusion, the data presented above indicate that the regulatory systems for REE-dependent alcohol dehydrogenases are not universally conserved and may be influenced by a variety of factors, in agreement with the wide divergence of the REE-dependent enzymes. Investigating the intrinsic regulation of these functions will help in understanding the causes for functional redundancies among alcohol dehydrogenases. To better understand such complex regulation, is vital to determine all the factors involved in the REE switch, which likely include membrane-bound, periplasmic and cytoplasmic factors.

## Development and application prospects for REE biotechnology

Proteins tend to be non-toxic, biodegradable, and they can be modified or evolved with relative ease to improve their properties (e.g. affinity, selectivity, and stability). Thus, to a certain extent, pursuing this venue may be more promising than relying on Ln^3+^ chelators for REE extraction, separation, and recycling [101]. At this point, protein modification and engineering so far mostly targeted LanM, considered as the most REE-selective protein [14, 15], thus potentially most industry-relevant [81]. LanM derived from *M. extorquens* or *H. quercus*, immobilized on agarose beads, magnetic nanoparticles, or natural structural proteins (e.g. elastin-like peptides), have successfully extracted REEs from a range of complex materials [14, 102]. Specific conformational changes in LanM may potentially profoundly affect its binding affinity for REEs. For example, LanM was transformed into DLanM, which could effectively bind up to six REEs and had remarkable selectivity, fast adsorption kinetics, and durable adsorption capacity [103]. A lanthanide-responsive fluorescent probe LaMP [33] and a REEs sensing variant LanTERN based on LanM’s unique EF-hand structure were also designed [104]. In addition to LanM, the newly discovered non-enzymatic protein LanP could also be isolated and purified for REE binding [16].

REEs also could be enriched and isolated from complex mixtures, and microorganisms could be modified through chemical modification, combined label surface display, and genetic engineering [5]. For example, by using sodium tripolyphosphates to chemically modify the surface of yeast, a new yeast that could achieve selective adsorption and concentration of REEs present at low concentrations, in the presence of multiple pollutants has been developed [25]. REE binding protein LanM was immobilized to the protein anchoring site CWP110 on the surface of *Yarrowia lipolytica* [105]. The modified yeast could efficiently and economically recover a broad spectrum of REEs from acidic wastewater. In addition, engineered *Escherichia coli* based on lanthanide-binding tags (LBT) had been constructed so that this variant could adsorb REEs [106]. Genes in strains considered to have the potential of bioleaching REEs have been studied through genetic engineering and other means [107]. Furthermore, recombinant *Escherichia coli* DH5α has also been developed, and *in situ* synthesis of seven REE amorphous nanoparticles ranging from La^3+^ to Gd^3+^ (but not Pm^3+^) was achieved [108]. Screening for microorganisms that could directly utilize or leach REEs through optimization of medium components or growth environment presents another strategy. For example, *M. extorquens* AM1 showed increased accumulation of Nd^3+^ through optimization of the medium used. Similar methods have been reported for the recovery of Gd^3+^ from medical waste [109].

For example, Samiappan et al. proposed to adjust the 3D structural stability of protein-metal complexes by artificially modifying the design of different metal binding sites/pockets to screen different metals [110]. Slope et al. designed a series of different binding sites in the synthetic protein sequence to achieve binding with a wider range of REEs [111]. Dykeman-Bermingham et al. presented a design to alter sequence-controlled polymers by altering sequence-controlled copolymers as REE chelators [112]. The structure and binding properties of the polymers were enhanced by changing the sequence patterns so that the biomacromolecules displayed different forms and functions. As a result, the binding affinities, abilities, and REE selectivity of the polymers were affected. An MDH that could dissolve REEs was purified from *M. extorquens* AM1 strain growing in a tailing environment, suggesting that MDH could be a selective adsorbent for extracting REEs [103]. In addition, artificial enzymes using REEs for multifunctional catalysis have been designed [112]. Current biotechnology for the recovery and purification of REEs still needs to overcome multiple technical challenges and be scaled for industrial production, to fully realize their potential in environmental protection and resource recovery.

## Conclusions and future prospects

**Figure 6:**
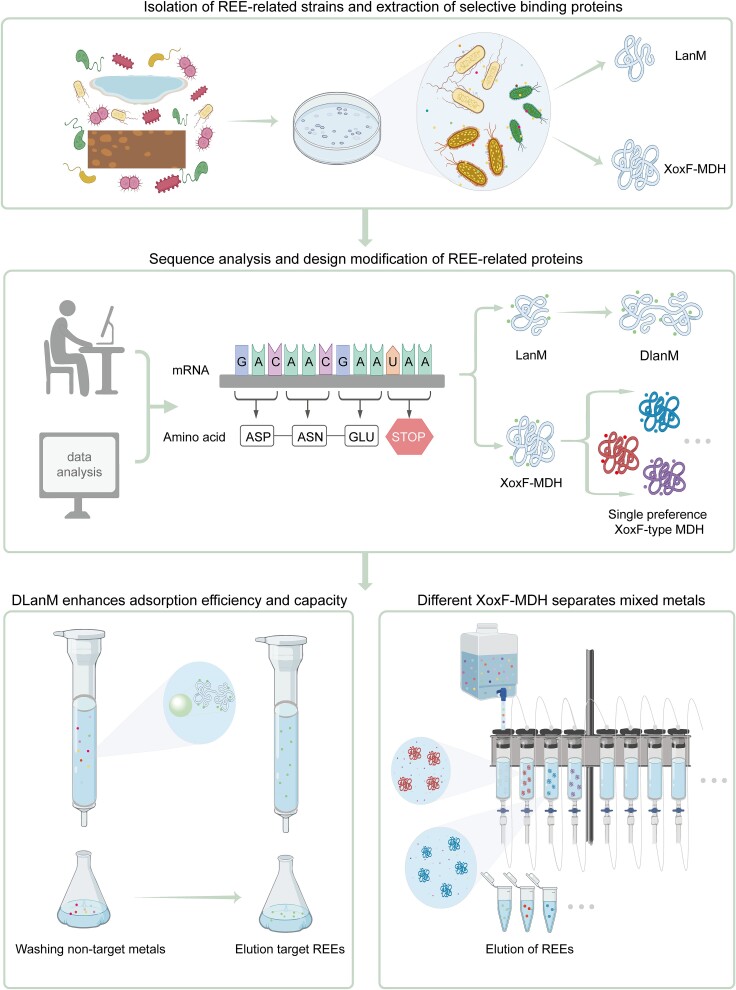
Schematic outlook on biomimetic or biological methods for mining, enrichment, and isolation of REEs related to REE-dependent proteins**. I**solation of REE-dependent strains from soil/water/mineral environments, and isolation and purification of REEs-related proteins (e.g. XoxF-MDH and LanM), can be modified in two ways, either by assembling the proteins into a double or multiplexed state, which would increase the efficiency of adsorption and separation of the REEs, or by substituting or adding or subtracting specific amino acids at the metal-binding site, or by using the protein in a biomimetic or biological way to enrich and separate REEs. To enhance the specificity of a protein for REE binding, we could modify the metal-binding site by adding or substituting specific amino acids, such as replacing or increasing the presence of residues like asp or Cys, which are known to coordinate REE ions effectively. This engineered selectivity would allow for efficient elution and separation of REEs, ensuring that only the desired metals are harvested or isolated. Such a design would be crucial for developing effective methods to separate mixed REEs, facilitating the sustainable extraction and purification of REEs.

The importance of REEs in industry and science underscores the continuing importance of exploring their extraction and utilization, expanding an already thriving field of research. Central to this work is the pursuit of efficient utilization and separation of pure REEs, which remains a key driver of future research and design. Although REE biotechnology development is booming, it appears that the ability of alcohol dehydrogenases and other molecules to bind REEs has not yet been fully exploited. In addition to existing emerging biotechnologies, the specific structures common among REE-binding biomolecules mentioned above suggest that it may be possible to design artificial biological macromolecules or active labels for the specific separation of REEs or their mixtures at structural levels such as metal-binding site interactions or active site-specific amino acid composition. Screening for biomolecules displaying high affinity for REEs in relevant microorganisms would be another effective approach. Inspired by structural differences, higher selectivity between REEs can be achieved by changing the geometry of relevant proteins. In addition, new REE binding motifs can be designed based on extant sequence information using molecular biology techniques such as site-directed mutagenesis and directed evolution (Fig. 6). Despite all the progress that is currently happening, the field of research applying alcohol dehydrogenases and other molecules with affinity to REEs at the industrial scale is still in its infancy. Similarly, the mechanisms of REE absorption and transport pathways are still not fully elucidated, prompting speculations on existence of yet undiscovered proteins and on their potential roles within cells. These unresolved questions highlight the current limitations in REE biotechnology. However, this field of research, having emerged only several years ago, is very active and growing dramatically. Thus, the prospects for significant breakthroughs, including in near future, are very optimistic.

## Data Availability

Data sharing does not apply to this article as no datasets were generated or analyzed during the current study. In addition, the data content used in this review is identified as a reference or data source (e.g. PDB database identifier in the legend).
